# Computer-Assisted Tracking of *Chlamydomonas* Species

**DOI:** 10.3389/fpls.2019.01616

**Published:** 2020-01-31

**Authors:** Alexandra M. Folcik, Timothy Haire, Kirstin Cutshaw, Melissa Riddle, Catherine Shola, Sararose Nassani, Paul Rice, Brianna Richardson, Pooja Shah, Nezamoddin Nazamoddini-Kachouie, Andrew Palmer

**Affiliations:** ^1^Department of Biomedical and Chemical Engineering and Sciences, Florida Institute of Technology, Melbourne, FL, United States; ^2^Department of Aerospace, Physics, and Space Sciences, Florida Institute of Technology, Melbourne, FL, United States; ^3^Department of Mathematical Sciences, Florida Institute of Technology, Melbourne, FL, United States; ^4^Department of Ocean Engineering and Marine Sciences, Florida Institute of Technology, Melbourne, FL, United States; ^5^Aldrin Space Institute, Florida Institute of Technology, Melbourne, FL, United States

**Keywords:** *Chlamydomonas reinhardtii*, automated tracking, algae motility, chemical biology, phototaxis, *Chlamydomonas moewusii*

## Abstract

The green algae *Chlamydomonas reinhardtii* is a model system for motility in unicellular organisms. Photo-, gravi-, and chemotaxis have previously been associated with *C. reinhardtii*, and observing the extent of these responses within a population of cells is crucial for refining our understanding of how this organism responds to changing environmental conditions. However, manually tracking and modeling a statistically viable number of samples of these microorganisms is an unreasonable task. We hypothesized that automated particle tracking systems are now sufficiently advanced to effectively characterize such populations. Here, we present an automated method to observe *C. reinhardtii* motility that allows us to identify individual cells as well as global information on direction, speed, and size. Nutrient availability effects on wild-type *C. reinhardtii* swimming speeds, as well as changes in speed and directionality in response to light, were characterized using this method. We also provide for the first time the swimming speeds of several motility-deficient mutant lines. While our present effort is focused around the unicellular green algae, *C. reinhardtii*, we confirm the general utility of this approach using *Chlamydomonas moewusii*, another member of this genus which contains over 300 species. Our work provides new tools for evaluating and modeling motility in this model organism and establishes the methodology for conducting similar experiments on other unicellular microorganisms.

## Introduction

The unicellular alga *Chlamydomonas reinhardtii* is a model organism for the study of flagellar motility, photosynthesis, and a variety of biotechnology applications among unicellular eukaryotes. This photoautotroph has minimal culture requirements, is genetically tractable, and has an extensive strain repository including numerous motility mutants (Luck et al., 1977; Huang et al., 1981; Huang et al., 1982a; Huang et al., 1982b; Kuchka and Jarvik, 1982; Segal et al., 1984; Kamiya, 1988; Barsel et al., 1988; Bloodgood and Salomonsky, 1989; Kuchka and Jarvik, 1987; Kamiya et al., 1991). Photo-, gravi-, and chemotaxis have all been associated with this organism, making it an ideal system for understanding how multiple inputs can be integrated to regulate motility in unicellular organisms. Prior efforts to quantify *Chlamydomonas* motility have largely focused on high-speed photographic evidence, which was then analyzed by manual tracking (Racey et al., 1981). However, such methods are time-consuming, prone to error, and unlikely to resolve multiple responses within a population. Moreover, algal cultures frequently reach densities ranging from 10^5^ to 10^7^ cells/ml. The results of a handful of tracks (<30) are unlikely to be an accurate reflection of the behavior of such a population. The ability to observe a larger sample size would also provide refined insight into the dynamic response range of this model organism rather than just an average.

Automated particle tracking in which individual constituents of a population, biotic or abiotic, are followed over time can yield data on particle speed, directionality, size, and other features of interest for understanding the behavior of the observed population. In the case of *C. reinhardtii*, we hypothesized that such approaches would be able to accommodate significantly more tracks, providing better models of behavior within a population of cells. Here, we report a new method to characterize motility in *C. reinhardtii*, which allows us to identify individual particles (cells) as well as gather population-wide information on speed as well as directionality. The proposed strategy requires only a microscope with a camera to collect images and utilizes a publicly available software package, the TrackMate plugin for ImageJ, for analysis (Schneider et al., 2012; Tinevez et al., 2017).

In the present study, we evaluated the impacts of light and nutrient availability on motility in wild-type *C. reinhardtii*, as well as characterizing a series of motility-deficient mutant lines. The ability to quantify the effects of such mutations provides a refined perspective on the impacts such mutations may have on these organisms. Our work provides a new tool for evaluating and modeling motility in this model organism. Furthermore, we confirm that the established methodology is able to characterize motility in another member of this genus, *Chlamydomonas moewusii*, supporting the broader utility of this approach for observing motility in other unicellular microorganisms which may have important roles in host–microbial associations and/or biotechnology.

## Materials and Methods

### Materials

Unless stated otherwise, all reagents were purchased from either Fisher Scientific or Sigma Aldrich.

### Algae Growth And Media

*C. reinhardtii* wild-type (cc124), *C. reinhardtii* mutants (see **Table 1**), and *C. moewusii* (formerly *Chlamydomonas eugametos*) were acquired from the Chlamydomonas Resource Center (http://www.chlamycollection.org/) (Luck et al., 1977; Huang et al., 1981; Huang et al., 1982a; Huang et al., 1982b; Kuchka and Jarvik, 1982; Segal et al., 1984; Kamiya, 1988; Barsel et al., 1988; Bloodgood and Salomonsky, 1989; Kamiya et al., 1991). Individual lines were maintained by streaking cultures on plates of Tris-acetate-phosphate (TAP) media with agar. Liquid cultures were prepared by growing lines individually in 25 ml of TAP media for 72 h under a 16:8-h day/night cycle (~13,800 W/m^2^) at 23°C on a shaker (~150 rpm) (Haire et al., 2018). Minimal media studies were performed by limiting the amount of available nitrogen by restricting the amount of NH_4_Cl, either to: low (50%, 400 mg/l) or NH_4_-free TAP media.

**Table 1:** Chlamydomonas *reinhardtii* motility mutants, their mutation types, and average speeds (±standard error) as compared to wild-type (cc124).

Strain ID	Mutation type^1^	Avg. speed^2^	% Wild-type	Reference
cc124	Wild-type	40.0 ± 4.5	100	–
cc125	Phototactic aggregation	46.0 ± 2.0	115	Pröschold et al., 2005
cc602 pf1	No radial spoke heads	2.8 ± 0.6	5	Luck et al., 1977
cc1026 pf3	Axonemal protein defects	9.0 ± 5.0	20	Huang et al., 1982b
cc1032 pf14	Lacks radial spokes	3.5 ± 0.1	6	Huang et al., 1981
cc1036 pf18	No central microtubules	1.5 ± 0.7	3	Bloodgood and Salomonsky, 1989
cc1926 uni1	Single flagellum cells	15.0 ± 3.0	38	Huang et al., 1982a
cc2228 oda1	Lacks outer dynein arms	11.0 ± 2.0	23	Kamiya, 1988
cc2288 lf2-4	Long flagella	32.0 ± 1.0	80	Barsel et al., 1988
cc2530 vfl2	0–6 flagella	4.0 ± 2.0	10	Kuchka and Jarvik, 1982
cc2670 ida4	Dynein arms	14.0 ± 2.0	35	Kamiya et al., 1991
cc2679 mbo1	Flagellar axoneme	3.4 ± 0.6	9	Segal et al., 1984
cc3663 shf1	Short flagella	22.0 ± 2.25	50	Kuchka and Jarvik, 1987

^1^Reference. ^2^Average speed expressed in terms of micrometers per second with standard error as determined by image analysis in this study.

### Video Acquisition

All motility data was acquired under minimal lighting in a darkroom to minimize background phototactic effects. Cell density was determined by fixing 200 µl aliquots of cells in 1.7% formaldehyde and manually counted using a hemocytometer at ×400. The remainder of the culture was incubated at 23°C in the dark for 30 min to reduce the impact of light on cell movement. Algae cultures were vortexed for ≈30 s to resuspend any settled algae. Aliquots of 30 µl were placed on a slide and viewed under an Olympus CH30 binocular microscope at ×100 magnification. Cells were allowed to settle to avoid artificial movement of the cells caused by convection currents in the liquid on the slide. ToupView software (www.touptek.com) was used to collect videos with an AmScope FMA050 fixed microscope camera. Videos were collected with a frame rate of 7.5 frames/s for approximately 30 s.

### Video Analysis

Videos were imported into Fiji for splitting and tracking analysis (Schindelin et al., 2012). Calibration was done using a micrometer ruler slide to determine pixel length. To analyze tracks, the pre-installed Fiji plugin TrackMate was utilized (Tinevez et al., 2017). Cell size was also determined through Fiji. The resulting files were saved in comma-separated values (CSV) format as “Spots in tracks statistics,” “Track statistics,” and “Links in tracks statistics.”

To determine the directionality of each track, the files “Spots in tracks statistics,” “Track statistics,” and “Links in tracks statistics” were combined using the statistical computing software R using the RStudio interface (www.rstudio.com) (R Development Core Team, 2016). This allowed for the creation of a new file in Tab Delimited Text format containing direction vectors of all cell tracks. From the combined R file, Rose plots were generated by dividing the slide viewing area into eight wedges and plotting the total fraction of cells in each section. Preliminary findings were confirmed using the Chemotaxis and Migration Tool (www.ibidi.com). Histograms of varying speeds within the population were generated by the data analysis package in Excel. Average speeds of tracks were determined using the “Track statistics” file. Units were converted to micrometers per second by dividing the time of the video by the number of image slices.

### Phototaxis Studies

Light intensity studies were completed using a Leica 13410311 Illuminator dissection scope leveled with the microscope platform. This allowed for the light to pass perpendicular to the slide across the sample. Light intensity settings were measured using Digital Luxometer. Cultures of 30 µl were loaded onto slides and exposed to the indicated intensity of light immediately before data collection.

## Results

### Particle Tracking

The digital equivalent of manual tracking across multiple video frames with hundreds of particles (cells) rapidly becomes computationally restrictive if the spatial coordinates of every possible combination has to be recorded from frame to frame over the period of these videos (30 s). This is an example of what is known as the ‘linear assignment problem’ and is well established in image analysis (Kong et al., 2013; Diem et al., 2015). However, this limit can be overcome by a variety of approaches. We utilized a publicly available software package, the TrackMate plugin for ImageJ, for analysis. In this package, videos are separated into individual frames, and the total number of particles, in this case cells of *C. reinhardtii*, in each frame are counted by identifying the edges using a Laplacian of Gaussian (LoG) detector, a common approach in image analysis (Leal Taixé et al., 2009; Kalaidzidis, 2009). TrackMate then matches particles across multiple frames using the Munkres–Kuhn (aka Hungarian) algorithm which identifies the most likely matches for the particles between frames and is also a well-established method for tracking multiple particles (Kong et al., 2013; Diem et al., 2015).

### Visualizing Cells and Quantifying Motility

**Figure 1:**
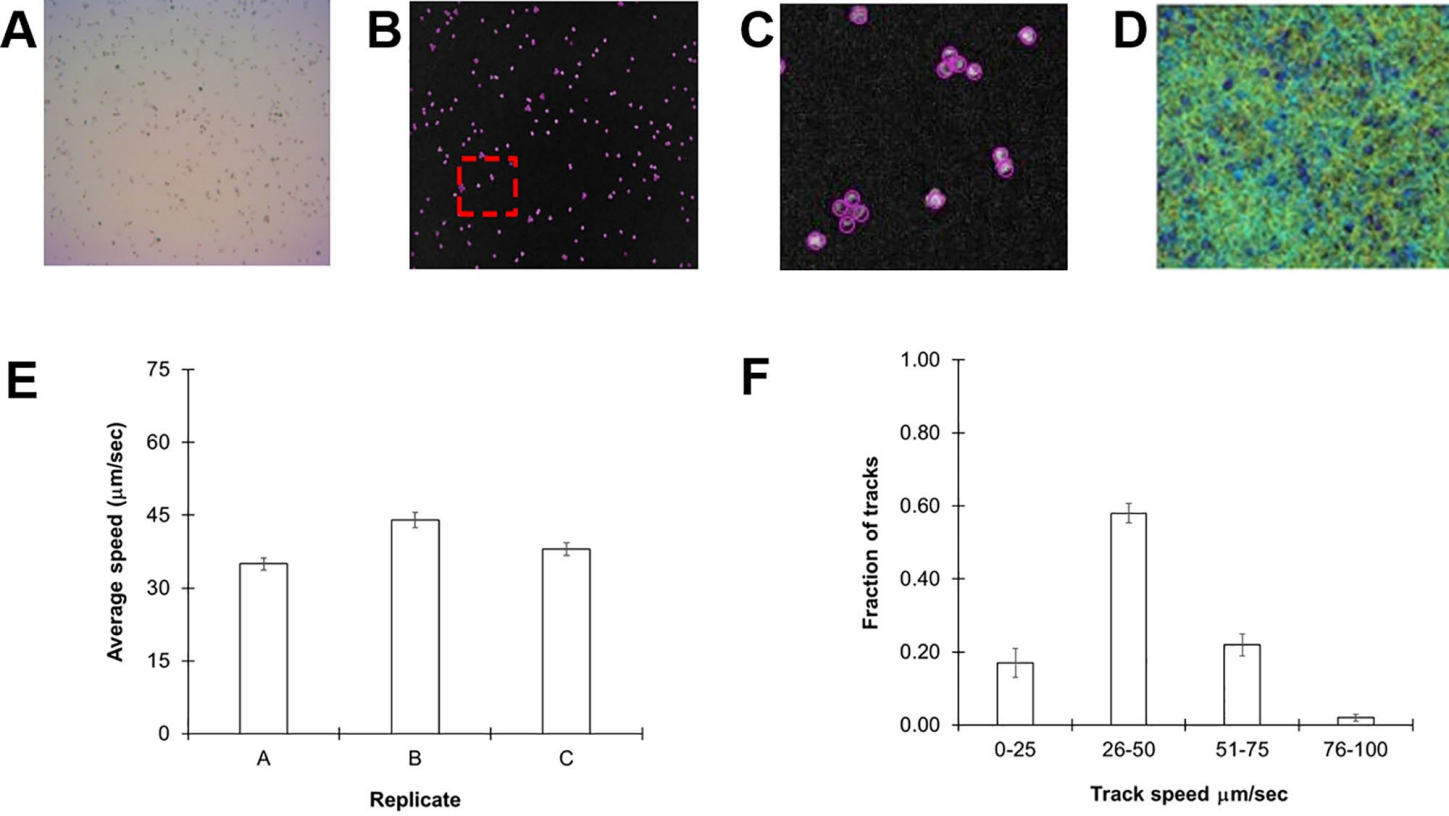
Monitoring *Chlamydomonas reinhardtii* motility by automated video tracking. **(A)** Image slice from cc124 72-h culture. **(B)** Isolated video frame/slice from ImageJ. **(C)** Magnification of image contained within red box of **(B)**, highlights individual spheres around tetrads and diads, underscoring the ability of the software to distinguish individual cells even in aggregates. **(D)** Heat map showing all tracks over a 30-s video from a 72-h culture. **(E)** Replicate study of 72-h-old cc-124 cultures. Each replicate was performed on a different day and is expressed as the average of three different samples recorded on that day, with error bars representing standard error. **(F)** Histogram showing distribution of tracks at different speeds from the same samples, with error bars representing the standard error.

We selected *C. reinhardtii* cc124, a common lab strain, for our initial experiments as it is the parent line for a number of motility mutants. Cultures were grown for 72 h in standard TAP media at room temperature to a density of 10^7^ cells/ml (see *Methods*). Thirty-second videos of 30 µl aliquots of cultures were acquired and subsequently processed and analyzed in ImageJ with the help of the TrackMate plugin. A representative image slice extracted from one of the videos is shown in **Figure 1A**. Each frame of the video is automatically separated into different slices and the particles (algal cells) counted (**Figure 1B**). *C. reinhardtii* is known to form motile aggregates such as diads and tetrads, which could skew the counting process. However, the software reliably distinguished individual members of this collection of cells (**Figure 1C**). From these videos, we were able to acquire 3,000–6,000 individual tracks for analysis over a 30-s period (see **Supplementary Video 1** ).

Cells were then tracked frame by frame to determine average speeds (**Figure 1D**). The average speed of our cc124 populations at 72 h was 40 ± 4.5 µm/s (**Figure 1E**). These findings are slower than some of those derived from previous studies, which can range from 80 to 200 µm/s (Racey et al., 1981; Marshall, 2009; Engel et al., 2011). However, close inspection of our track speeds confirmed an upper rate of ≈93 µm/s, in the range of these previously reported values. A histogram of speed distributions in this population confirmed that >50% of the total tracks for each sample (3,000–6,000 tracks/video) were within the 31- to 60-µm/s range (**Figure 1F**), consistent with the average speed.

### Using *Chlamydomonas* Mutants to Observe Changes in Motility

**Figure 2:**
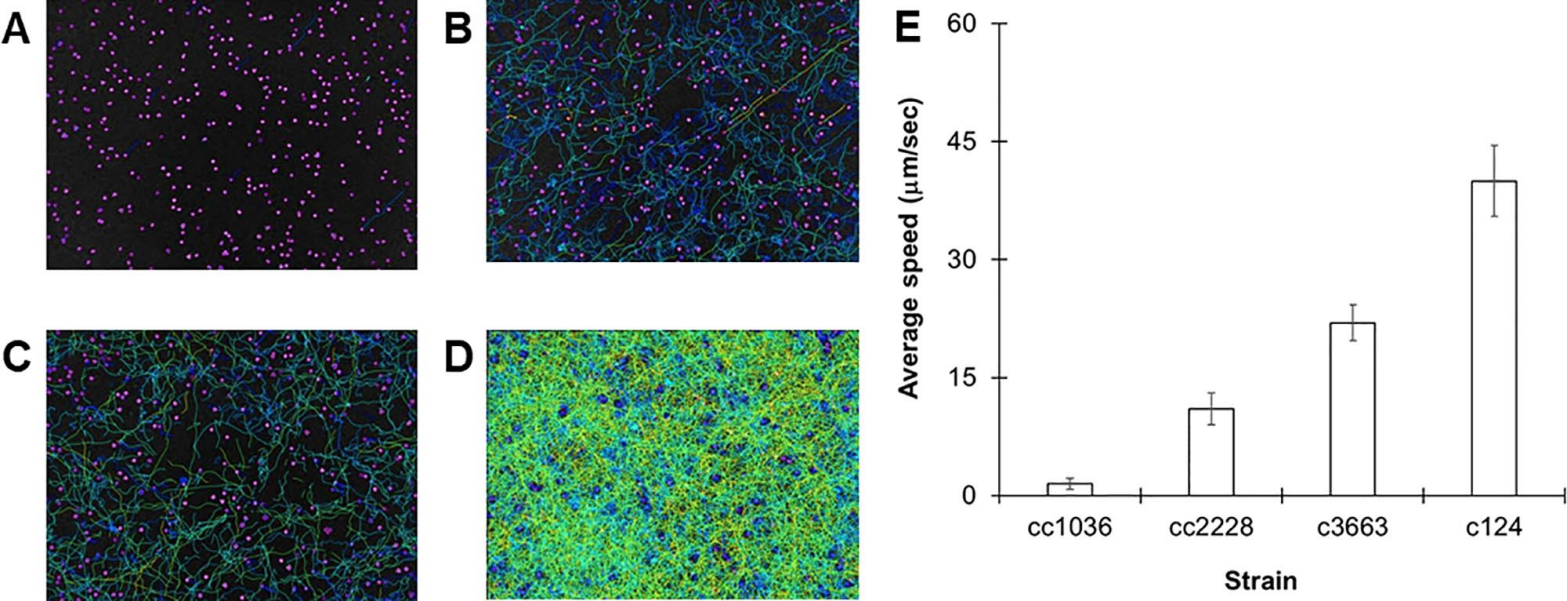
Using *C. reinhardtii* motility mutants to observe different populations. **(A**–**D)** Heat maps showing all tracks over 30-s videos from 72-h cultures for cc1036 **(A)**, cc2228 **(B)**, cc3663 **(C)**, and cc124 **(D)** strains. **(E)** Average speed in micrometers per second for the indicated strain of *C. reinhardtii*. Results are expressed as the mean of three videos, with error bars representing standard error.

A large assortment of motility mutants are available for *C. reinhardtii* which have helped elucidate the mechanisms of flagellar motility. Unfortunately, the characterization of such mutants is generally limited to relative statements, i.e., “slower” or “extremely slow,” rather than being quantified. In order to confirm the robustness of our tracking approach, we evaluated the swimming speeds of several mutant cell lines of *C. reinhardtii*. We initially investigated three specific mutant strains: cc1036, a non-motile line, as well as cc2228 and cc3663, both motility-deficient lines. Video analysis confirmed that the total number of tracks for each sample increased in the following order: cc1036, cc2228, cc3663, and finally cc124, respectively (**Figures 2A–D**). The average speed of the mutant lines ranged from ≈1 µm/s (cc1036) to 20 µm/s (cc3663) based on a minimum of 1,000 tracks/video for each mutant (**Figure 2E** and **Table 1**).

Our results establish lines cc1036, cc2228, and cc3663 as examples of 0%, 25%, and 50% motility lines, respectively, when compared to the wild-type cc124 strain. A sample video for cc3663 is provided as **Supplementary Video 2** (see **Supplementary Material**). Closer inspection of the 25% mutant (cc2228) showed that a small fraction of these mutants (<0.01%) were actually able to obtain speeds in the 21- to 40-µm/s category, with a maximal speed of 30 µm/s. The ability to obtain this information provides valuable insight into the stochastic nature of cell populations, but may also be used for identifying unique behaviors and compensatory mutations which may arise. Using this method, we obtained the average cell speeds for 12 different *C. reinhardtii* mutant lines (**Table 1**). We note that the cc125 line, which is deficient in phototaxis, actually moves slightly faster than the cc124 strain (115%, *p* = 0.05, as determined by Student’s *t* test). In addition to the successful characterization of swimming speeds in mutant lines, this approach appears to have even broader utility as we were also able to characterize the swimming speed of a closely related species, *C. moewusii* (48 +/– 6 µm/s).

### Characterizing Mixed Populations

**Figure 3:**
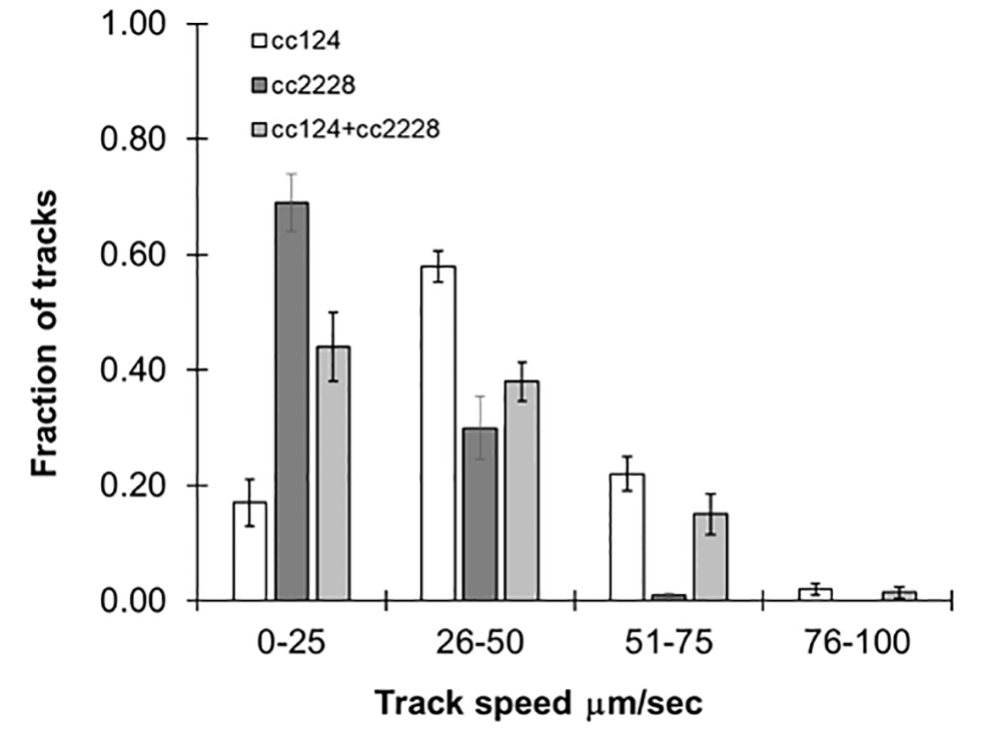
Following mixed populations by video tracking. Histograms showing the distribution of speeds for 72-h cultures of cc124 (wild type), cc2228 (25% motility), and a combined culture of both. Results are the mean of three videos, with error bars representing standard error.

Our method was able to measure swimming speeds in populations which converged around a single maximum number of tracks. However, it should also be robust enough to handle mixed populations of varying speeds. In order to evaluate this, we combined equal concentrations of 72-h cultures of cc124 and cc2228, a mutant with 25% motility of wild-type. As seen in **Figure 3**, the distribution of track speeds for mixed cultures was distinct from either of the cultures for each line individually, with an average speed of 36 ± 2.5 µm/s. These findings underscore the ability of this approach to observe variations within the population.

### The Effects of Culture Conditions on Speed

**Figure 4:**
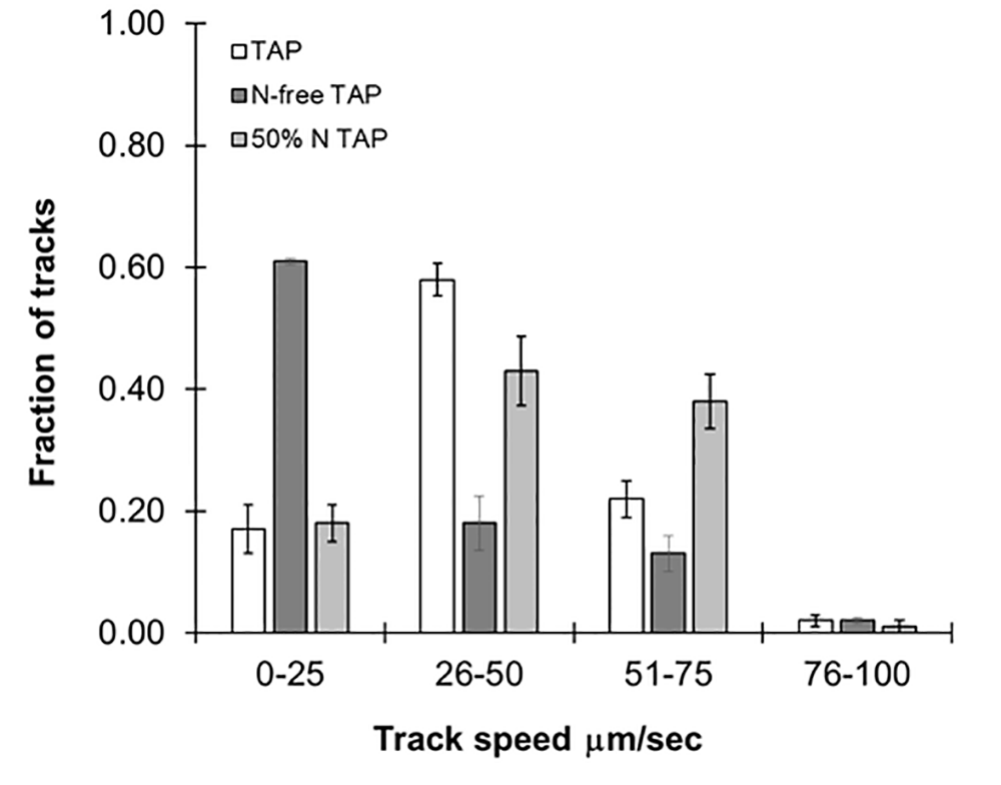
Effects of different media types on motility. Histograms showing distribution of speed across cc124 cultures grown in Tris-acetate-phosphate (TAP), N-free TAP, or 50% N-TAP, respectively. Results are the mean of three videos, with error bars representing standard error.

While specific mutations impact swimming speed, it is changing environmental conditions such as nutrient availability and light which influence speed and directionality of motility in the wild type. As previously stated, this particular strain of *C. reinhardtii* is known to be sensitive to nitrogen availability, and we tested the effect of this on swimming speed. We prepared reduced nitrogen TAP by limiting the amount of NH_4_Cl added to the media to either 50% or no NH_4_Cl. As shown in **Figure 4**, a fraction of the algae cultured in 50% NH_4_Cl TAP (≈40%) were observed in the 51- to 75-µm/s range, while in regular TAP only 20% of the cultures reached this range. We propose that this unexpected increase in swimming speed is to support the search for new nitrogen sources in this nutrient-depleted environment. This was in stark contrast to cells cultured in NH_4_Cl-free TAP, which significantly reduced motility, not surprising given its importance in flagellar motility and development (Engel et al., 2012). We note that a small fraction of these cultures (<5%) seemed unaffected by the nitrogen availability differences in these media, suggesting some compensatory mechanisms may be at work. Ongoing studies in our lab are further exploring media effects on swimming speeds in this model organism.

### Visualizing Phototaxis in cc124

**Figure 5:**
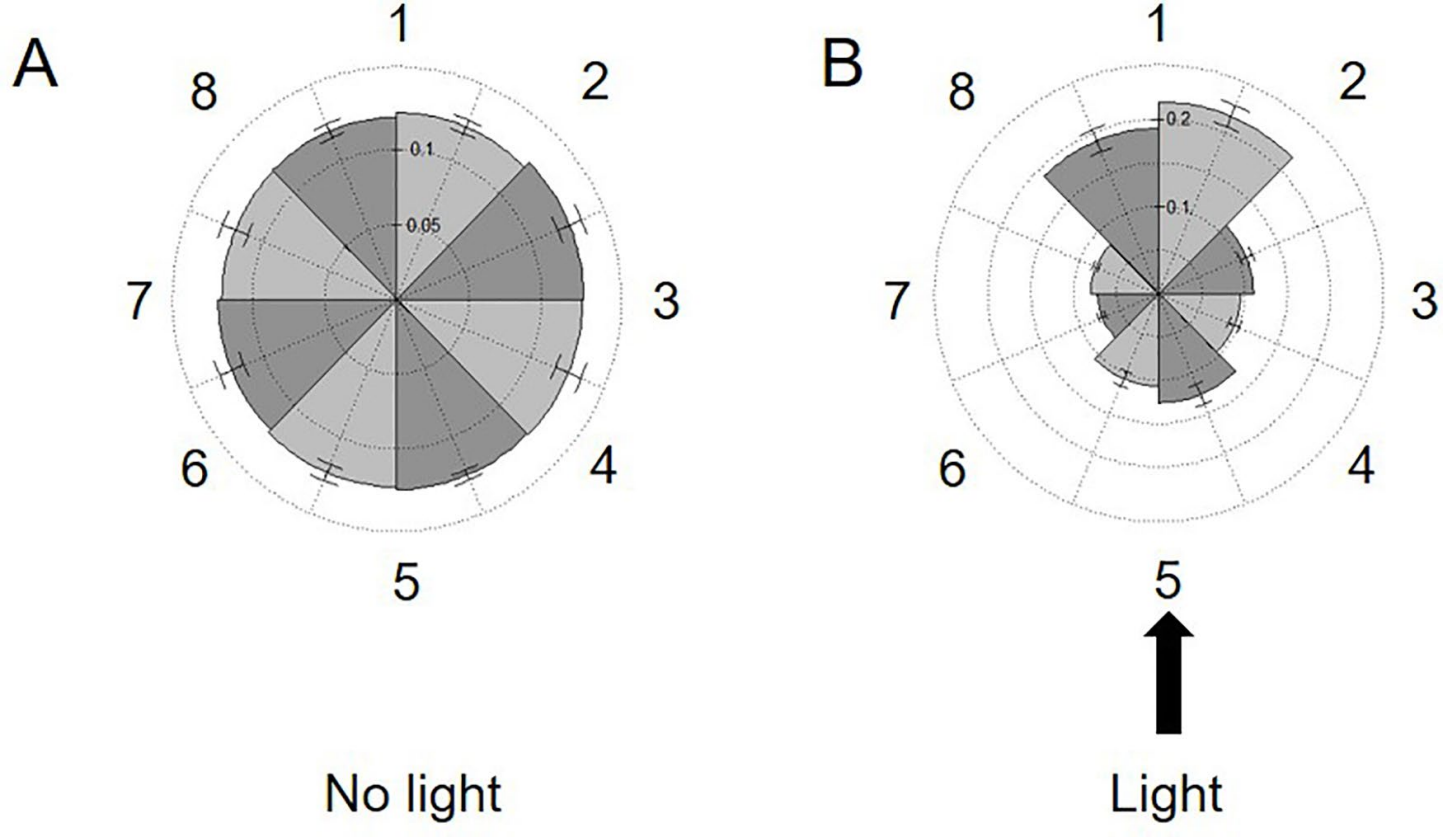
Measuring phototaxis across the imaging area. Rose plots dividing the viewing area into eight sections (numbered left to right *1*–*8*) are populated with the spatial coordinates of all tracks in 30-s videos of 72-h cultures of *C. reinhardtii* cc124 either: **(A)** without or **(B)** in the presence of a 75,172-W/m^2^ external light source. This light source was placed perpendicular to the slide surface (at section 5). The fraction of tracks in each section is noted on the internal axes/spokes. Results are expressed as the average of three experimental replicates, with error expressed as standard deviation.

The studies above confirm the utility of this approach to obtain population-level resolution of variations in swimming speed in response to mutation or changing environmental conditions. However, the same method used to determine speed also provides spatial coordinates for each track, allowing us to measure overall changes in the directionality of our samples. In this approach, the coordinates of each track are used to determine where the samples reside within the image. The image space is then divided into eight distinct sectors and populated accordingly. Analysis of our 30-s videos of cc124 in regular TAP media confirmed that the cells were uniformly distributed across the slide, establishing that there is no directionality bias (artifact) in our technique which might impact our study (**Figure 5A**).

Phototaxis is a commonly observed phenomenon in the cc124 line. Cells will move into the path of light, but also away from the source, presumably to minimize photosynthetic stress. We next sought to exploit this response to see if we could observe population-wide directionality changes utilizing our approach (Harris et al., 2013). Phototaxis was induced by placing a narrow beam of white light (≈75,000 W/m^2^) perpendicular to the focal plane of the slide. As shown in **Figure 5B**, within 5 min of light exposure, the bulk of the cells in the sample oriented into the path of the light but also into the three sectors furthest from the light source. Surprisingly, there was no increase in swimming speeds within the population in response to this stimulus. These findings confirm the ability of our approach to detect changes in directionality across the window provided by the microscope camera (8.19 mm diagonally across the camera window). While preliminary, this experiment confirms the ability of this approach to observe responses to stimuli within the experimental environment (i.e., the microscope slide). Future experiments will explore the potential for chemotaxis across the surface area of the slide.

## Discussion

Automated image analysis provides us with an opportunity to observe and characterize population-level responses, overcoming the limits to statistical resolution and bias associated with manual measurements. In the present study, we have developed a method requiring no custom-designed equipment or software, only a microscope, camera, and the free software package Fiji to analyze the motility of *C. reinhardtii*. Employing this method, we were able to observe differences in swimming speeds between the wild-type strain and several motility mutants, providing quantitative values to these mutants for the first time. In addition to motility mutants, we showed that our approach was sensitive to changes in media composition such as nitrogen availability. As expected, the removal of NH_4_Cl from TAP seriously compromised motility, but unexpectedly, a 50% reduction in NH_4_Cl resulted in an increase in the swimming speed of a portion (20%) of the cells. We propose that this rate increase facilitates the search for new nitrogen sources under nitrogen-limited conditions.

When coupled to the microplate-based methods we have already developed for investigating the growth and viability of this microorganism, we are now prepared to thoroughly explore and characterize the impacts of changing environmental conditions and/or mutation on multiple aspects of *Chlamydomonas* biology (Haire et al., 2018). Indeed, our ability to measure swimming speeds in *C. moewusii* strongly supports the utility of this approach for understanding motility in this important genus of over 300 species. While focused on this model unicellular eukaryote, the approaches outlined here should be easily exportable to other genera, potentially providing new insights into microbial ecology, modes of pathogenesis, and other aspects of microbial behavior.

## Data Availability Statement

The datasets generated for this study are available on request to the corresponding author.

## Author Contributions

AF performed all phototaxis assays and mutant characterizations including experiments and analysis. TH contributed to phototaxis assays, identified mutant strains to be utilized, and assisted in methods development. KC performed all variable growth media studies and analysis. PS obtained the swimming speeds of *C. moewusii*. MR, CS, SN, and NN-K all participated in the initial development of the method. PR and BR helped simplify the method and validate the results presented herein. AP provided all resources and research oversight for the project.

## Conflict of Interest

The authors declare that the research was conducted in the absence of any commercial or financial relationships that could be construed as a potential conflict of interest.
